# Achieving Exceptional Cochlea Delineation in Radiotherapy Scans: The Impact of Optimal Window Width and Level Settings

**DOI:** 10.7759/cureus.37741

**Published:** 2023-04-17

**Authors:** Nandan M Shanbhag, Abdulrahman Sulaiman Bin Sumaida, Mohammad Saleh

**Affiliations:** 1 Department of Oncology/Palliative Care, Tawam Hospital, Al Ain, ARE; 2 Department of Oncology/Radiation Oncolgy, Tawam Hospital, Al Ain, ARE; 3 Department of Oncology, Tawam Hospital, Al Ain, ARE

**Keywords:** accurate delineation, x-ray computed radiographic image interpretation, inner/anatomy and histology image segmentation tomography, computer-assisted medical imaging radiology ear, computer-assisted radiographic image enhancement three-dimensional imaging image interpretation, cochlea computed tomography (ct) scan image processing

## Abstract

Introduction

Radiation therapy (RT) aims to maximize the dose to the target volume while minimizing the dose to organs at risk (OAR), which is crucial for optimal treatment outcomes and minimal side effects. The complex anatomy of the head and neck regions, including the cochlea, presents challenges in radiotherapy. Accurate delineation of the cochlea is essential to prevent toxicities such as sensorineural hearing loss. Educational interventions, including seminars, atlases, and multidisciplinary discussions, can improve accuracy and interobserver agreement in contouring. This study seeks to provide radiation oncology practitioners with the necessary window width and window level settings in computed tomography (CT) scans to accurately and precisely delineate the cochlea, using a pre-and post-learning phase approach to assess the change in accuracy.

Methods and materials

The study used the ProKnow Contouring Accuracy Program (ProKnow, LLC, Florida, United States), which employs the StructSure method and the Dice coefficient to assess the precision of a user's contour compared to an expert contour. The StructSure method offers superior sensitivity and accuracy, while the Dice coefficient is a more rudimentary and less sensitive approach. Two datasets of CT scans, one for each left and right cochlea, were used. The author delineated the cochlea before and after applying the proposed technique for window width and window level, comparing the results with those of the expert and general population. The study included a step-by-step method for cochlea delineation using window width and window level settings. Data analysis was performed using IBM SPSS Statistics for Windows, Version 26.0 (Released 2019; IBM Corp., Armonk, New York, United States).

Results

The implementation of the proposed step-by-step method for adjusting window width and window level led to significant improvements in contouring accuracy and delineation quality in radiation therapy planning. Comparing pre- and post-intervention scenarios, the author exhibited increased StructSure scores (right cochlea: 88.81 to 99.15; left cochlea: 88.45 to 99.85) and Dice coefficient scores (right cochlea: 0.62 to 0.80; left cochlea: 0.73 to 0.86). The author consistently demonstrated higher contouring accuracy and greater similarity to expert contours compared to the group's mean scores both before and after the intervention. These results suggest that the proposed method enhances the precision of cochlea delineation in radiotherapy planning.

Conclusion

In conclusion, this study demonstrated that a step-by-step instructional approach for adjusting window width and window level significantly improved cochlea delineation accuracy in radiotherapy contouring. The findings hold potential clinical implications for reducing radiation-related side effects and improving patient outcomes. This study supports the integration of the instructional technique into radiation oncology training and encourages further exploration of advanced imaging processing and artificial intelligence applications in radiotherapy contouring.

## Introduction

Radiation therapy (RT) aims to deliver the maximum dose to the target volume while minimizing the dose to the organs at risk (OAR) [1]. The accurate delineation of these OARs is critical in defining the borders of unaffected organs and the clinical target. This is important because large areas exposed to radiation can have significant side effects, while a missed target volume may result in inadequate local control and adversely affect overall survival [2]. While the target volume may vary throughout treatment, the organs at risk generally do not change anatomically, but preserving their function is crucial to maintaining a good quality of life for the patient [3]. Therefore, precise contouring is essential to achieving optimal treatment outcomes with minimal side effects. Modern radiation therapy techniques, such as intensity-modulated radiation therapy (IMRT) and volumetric arc therapy (VMAT), can further improve the precision and accuracy of radiation delivery while minimizing harm to healthy tissues and organs [4].

The head and neck regions are challenging for radiotherapy due to their complex anatomy and the potential for artifacts and image discrepancies between diagnostic computed tomography (CT) and CT simulation images, which are used in most linear accelerator (LinAc) centers for delineation of the target and the organs at risk. One of the organs at risk, the cochlea, needs to be accurately delineated, as one of the toxicities of higher doses to the organ is sensorineural hearing loss, in addition to tinnitus, pain, and otitis media [5].

The cochlea is a critical component of the auditory system and plays a central role in hearing. It is a spiral-shaped structure located in the inner ear and is responsible for converting sound waves into electrical signals transmitted to the brain. The vestibular duct is located in the uppermost part of the cochlea and is surrounded by the vestibular membrane. In contrast, the tympanic duct is in the middle and surrounded by the basilar membrane. The spiral canal, also referred to as the cochlear duct, is the lowermost part of the cochlea and is surrounded by the spiral ligament [6]. The cochlea is lined with hair cells, which are specialized sensory cells that are responsible for detecting sound waves and converting them into electrical signals. The hair cells are organized in a tonotopic manner, meaning that different regions of the cochlea are sensitive to different sound frequencies. This tonotopic organization allows the cochlea to perform frequency analysis and play a critical role in our ability to hear and distinguish different sounds [7].

The variation in volume delineation in radiotherapy treatments is a significant issue, affected by imaging modality resolution and inter- and intra-observer variability. To reduce contouring errors and improve the accuracy of treatment, educational interventions can be implemented. These interventions can include anatomy lectures, the completion of contouring modules, peer review of contoured volumes, seminar series, anatomy or region of interest atlases, one-on-one training, offline simulations, and protocols. Multidisciplinary discussion sessions with radiation therapists (RTs), radiation oncologists (ROs), and radiation oncology medical physicists (ROMPs) can also be beneficial in improving the understanding and accuracy of target volume delineation. Studies have shown that educational interventions, such as seminar series and atlases, can increase accuracy and improve the interobserver agreement. The use of image-guided radiotherapies, such as cone beam computed tomography (CBCT), has increased the complexity of RT tasks, and educational interventions can improve knowledge and accuracy in identifying soft tissue structures [8].

Several techniques have been used to spare the cochlea from head and neck radiation. For example, in a study that aimed to create guidelines for radiation oncologists to accurately contour the inner and middle ear on treatment planning CT scans, the authors reviewed the CT scans of 15 patients who underwent radiotherapy and used anatomic landmarks to contour the middle ear, cochlea, and vestibular apparatus. The study found that the mean volume of the middle ear was 0.58 cm^3^, the vestibular apparatus was 0.44 cm^3^, and the cochlea was 0.14 cm^3^. The maximum axial dimension across the contour was 1.57 cm for the middle ear, 1.10cm for the vestibular apparatus, and 0.69 cm for the cochlea [9].

The majority of existing atlases and contouring guidelines in radiation oncology are based on anatomical landmarks and structures but lack specific instructions on the appropriate window width (WW) and window level (WL) settings in CT scans to visualize the cochlear apparatus. The objective of this study is to provide radiation oncology practitioners with the necessary range of WW and WL settings in CT scans to accurately and precisely delineate the space occupied by the cochlear apparatus. This study utilizes a pre- and post-learning phase to determine the change in accuracy for delineating the cochlea once the WW and WL are implemented.

## Materials and methods

The authors used the online platform, the ProKnow Contouring Accuracy Program (ProKnow, LLC, Florida, United States), which employs two distinct methodologies to appraise the precision of a user's contour to the "gold standard" or expert contour. These approaches encompass the StructSure method (powered by StructSure™ by Standard Imaging, Inc., Middleton, Wisconsin, United States, US Patent 8,081,813) and the Dice coefficient [10].

StructSure method (heightened sensitivity)

The StructSure method, a patented technique initially published in 2012, yields an accuracy score between 0 and 100 [11]. This method utilizes a sophisticated three-dimensional algorithm that compares the volumes of both the user's and expert's contours, subsequently penalizing errant voxels according to their respective distance errors. Errant voxels can manifest as either "missing" or "extra." If an errant voxel is situated within a designated forgiveness region, typically 1 mm, no penalties are incurred. The ultimate score is computed as follows: 100 * [# expert voxels - sum (penalties, over all voxels)] / [# expert voxels]. The StructSure method exhibits superior sensitivity compared to the Dice coefficient.

Dice coefficient (diminished sensitivity)

The Dice coefficient quantifies the degree of overlap between two distinct volumes (or areas in two dimensions) and spans from 0.00, indicating no overlap, to 1.00, signifying perfect overlap. However, the Dice coefficient often lacks sufficient sensitivity for radiation therapy contouring applications, as it fails to differentiate between substantial and trivial voxel errors. The Dice coefficient is ascertained using the following formula: Dice coefficient = (2 * |V1 ∩ V2|) / (|V1| + |V2|).

Here, V1 and V2 denote the calculated volumes of the expert and user contours, respectively. The Dice coefficient will invariably exceed 0.00, provided any overlap exists between the expert's and the user's contour volumes [12].

In summation, the StructSure method offers superior sensitivity and accuracy in evaluating contour precision in radiation therapy, while the Dice coefficient constitutes a more rudimentary and less sensitive approach that solely measures the extent of overlap between two contour volumes [13].

Delineation of the cochlea

Two CT datasets, one each for the left and right cochlea, were used for this study. Both datasets were preloaded in the online contouring platform and were non-contrast CT scans of the head and neck with 2 mm slice thickness. The delineation of the cochlea was done by the author before and after applying the proposed technique for window width and window level, and the results were analyzed. The author's contours were compared with those of the expert and the general population. As of the time of this publication, a total of 863 individuals had participated in delineating the left cochlea and 772 in delineating the right cochlea.

A step-by-step description of the method used by the author to delineate the cochlea using the window width and the window level is described in detail below:

Step 1: Set the window width to 400 and the window level to 400. This ensures that only the skull bone is visible and the soft tissue is not seen. This is a key step to identify the empty, dark space anterior to the internal acoustic meatus, which is the cochlea.

Step 2: Commence superior to the auricle and traverse caudally until the internal acoustic meatus becomes distinctly discernible.

Step 3: Trace the course of the internal acoustic meatus toward the inner ear. Situated anterior to the internal acoustic meatus lies an empty, dark space (hypodense), encompassing the cochlea.

Step 4: Delineate the dark space. Progressively navigate in the caudal and cranial directions and outline the residual cross-sectional profiles of this dark space.

Step 5: Set the window depth to 2500 and the window level to 400 (bone window), which makes the soft tissue visible in addition to the bone and confirms the contours.

The data were analyzed using IBM SPSS Statistics for Windows, Version 26.0 (Released 2019; IBM Corp., Armonk, New York, United States) [14].

## Results

Explaining the scoring system 

A low or negligible StructSure score does not necessarily indicate a complete omission of the target organ. To evaluate the degree of overlap with the gold standard structure, the Dice coefficient (DC) should be considered. A positive DC value implies that a portion of the organ has been accurately delineated. As the DC value increases, nearing its maximum of 1.00, the overlap between the contoured and gold standard structures becomes more substantial.

The StructSure score is a highly sensitive metric, detecting all discrepancies and penalizing based on the magnitude of deviation between the delineated structure and the gold standard for each erroneous volume element. This score has demonstrated its utility in estimating potential dosimetric consequences in contemporary radiation treatment planning. In simple terms, the higher the scores, the better the contours, and the closer it is to the expert contour.

Dataset 1: right cochlea delineation

Author Compared to the Expert

The author's score before and after the implementation of the step-by-step instructions is given in Table 1. 

**Table 1:** The table presents a comparison of two sets of measurements, "pre-intervention" and "post-intervention", pertaining to regions of interest (ROIs) 1 and 2, alongside relevant evaluation metrics for radiotherapy contouring of the right cochlea. [10]

Measurement	Pre-intervention	Post-intervention
ROI 1 Volume (cc)	0.12	0.12
ROI 2 Volume (cc)	0.09	0.08
Matching Volume (cc)	0.06	0.08
Extra Volume (cc)	0.03	0.00
Missing Volume (cc)	0.06	0.04
Forgiven Volume (Extra) (cc)	0.02	0.00
Forgiven Volume (Missing) (cc)	0.03	0.04
Dice Coefficient	0.62	0.80
StructSure™ Score	88.81	99.15
Forgiveness Region (mm)	1.00	1.00
Penalty Slope (voxel penalty/mm)	0.50	0.50
Max Distance Threshold (mm)	10.00	10.00

The Table 1 presents a comparison of two sets of measurements, "pre-intervention" and "post-intervention", of regions of interest (ROIs) 1 and 2, alongside relevant evaluation metrics for radiotherapy contouring. The data is described as follows:

ROI 1 and ROI 2 volumes: The volumes of the two ROIs are presented in cubic centimeters (cc) for both the "pre-intervention" and "post-intervention" scenarios. ROI 1, which is the expert's contour, maintains a consistent volume of 0.12 cc, whereas ROI 2, which is the author's delineation of the cochlea, exhibits a slight reduction from 0.09 cc to 0.08 cc.

Matching, extra, and missing volumes: The matching volume, representing the overlap between the expert contour and the author-delineated structure, increases from 0.06 to 0.08 cc. The extra volume, which refers to the delineated volume outside the expert contour, decreases from 0.03 cc to 0.00 cc. The missing volume, corresponding to the undelineated portion of the expert contour, decreases from 0.06 cc to 0.04 cc.

Forgiveness metrics: The forgiven volumes for extra and missing portions are presented separately. For the extra volume, forgiven values decrease from 0.02 cc to 0.00 cc, while forgiven values for the missing volume remain consistent at 0.04 cc.

Dice coefficient: This metric reflects the accuracy of the delineated structure to the expert's delineation. An increase from 0.62 to 0.80 indicates enhanced overlap between the structures.

StructSure score: This sensitive metric gauges the overall quality of the delineated structure, with higher scores reflecting greater accuracy. The score improves significantly from 88.81 to 99.15.

Forgiveness region, penalty slope, and maximum distance threshold: These parameters remain constant in both scenarios. The forgiveness region, which determines the acceptable deviation from the expert contour, is set at 1.00 mm. The penalty slope, representing the voxel penalty per millimeter, is set at 0.50. Lastly, the maximum distance threshold, beyond which the penalty is maximized, is established at 10.00 mm.

Overall, the table demonstrates improvements in contouring accuracy and delineation quality in the "post-intervention" scenario which is the implementation of the window width and window level compared to the "pre-intervention" scenario, as evidenced by the increased Dice coefficient and StructSure™ score.

**Figure 1:**
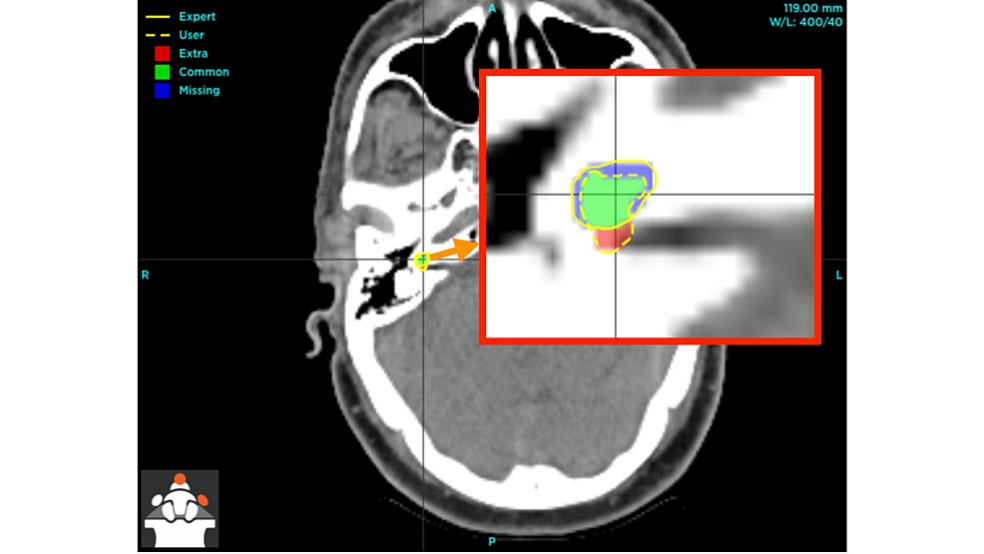
Pre-intervention discrepancies in right cochlea delineation The inset, indicated by an orange arrow, displays an enlarged perspective of the right cochlea, highlighting both the user (author) and expert contours as well as the regions of overlap and discrepancy between the two delineations [10].

**Figure 2:**
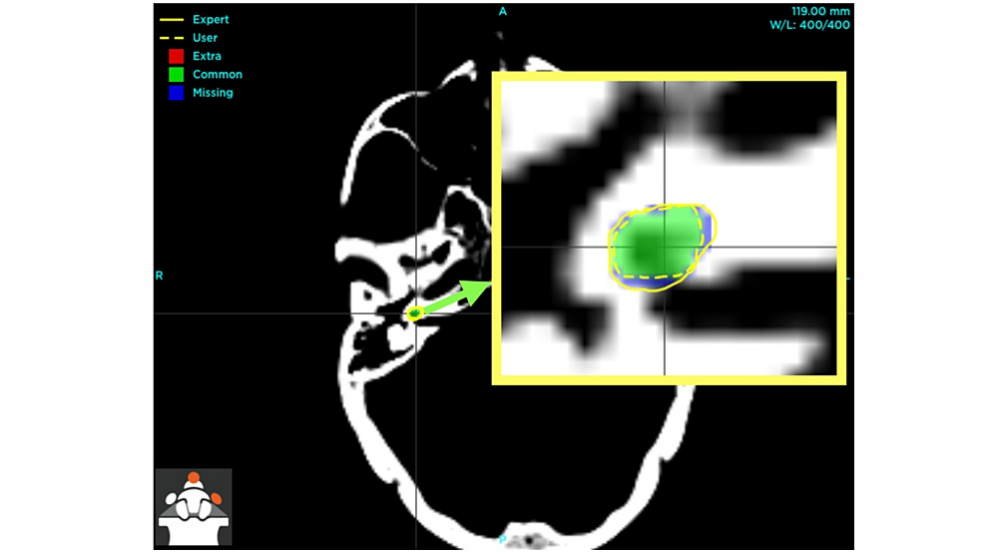
Post-intervention improvement in right cochlea visualization with optimized window width and level The inset, indicated by a green arrow, displays an enlarged perspective of the right cochlea, highlighting both the user (author) and expert contours, as well as the regions of overlap and discrepancy between the two delineations [10].

The discrepancies observed before the implementation of the outlined measures are evident (Figure 1). These images distinctly showcase the enhancement in the demarcation of the right cochlea following the execution of the prescribed steps, particularly the adjustment of window width and window level to effectively discern the hypodense region anterior to the internal auditory meatus (Figure 2).

Author Compared to the Population

A total of 772 participants (including the author) registered and executed the delineation exercise. We do not know the technique used by the 771 participants for the initial contours and later contours. As in the author's case, the initial contours (pre-intervention) were delineated with a window width of 400 and a window level of 40, while the later contours (post-intervention) were delineated after the implementation of the step-by-step instructions depicted in this study.

Table 2 compares the pre-and post-intervention StructSure and Dice scores for the group and the author.

**Table 2:** A comparison of the author's performance with the group [10]

Metric	Group Pre-Intervention	Group Post-Intervention	Author Pre-Intervention	Author Post-Intervention
StructSure Score	43.90 (mean)	91.62 (mean)	88.81	99.15
Dice Score	0.39 (mean)	0.75 (mean)	0.615	0.803

A comparison of the author's performance with that of the group reveals that the pre-intervention StructSure score (88.81) is notably higher than the group's mean pre-intervention score (43.90). Following the intervention, the StructSure score (99.15) remains higher than the group's mean post-intervention score (91.62), indicating consistent superior performance in comparison to the group.

Similarly, the author's pre-intervention Dice score (0.615) surpasses the group's mean pre-intervention score (0.39). After the intervention, the Dice score (0.803) exceeded the group's mean post-intervention score (0.75), suggesting that the author's delineation accuracy remained higher than the group's average.

Dataset 2: left cochlea delineation

Author Compared to the Expert

The author's score before and after the implementation of the step-by-step instructions is given in Table 3. 

**Table 3:** The table presents a comparison of two sets of measurements, "pre-intervention" and "post-intervention", pertaining to regions of interest (ROIs) 1 and 2, alongside relevant evaluation metrics for radiotherapy contouring of the left cochlea. [10]

Measurement	Pre-Intervention	Post-Intervention
ROI 1 Volume (cc)	0.14	0.14
ROI 2 Volume (cc)	0.12	0.15
Matching Volume (cc)	0.10	0.12
Extra Volume (cc)	0.03	0.02
Missing Volume (cc)	0.04	0.02
Forgiven Volume [Extra] (cc)	0.03	0.02
Forgiven Volume [Missing] (cc)	0.01	0.02
Dice Coefficient	0.73	0.86
StructSure™ Score	88.45	99.85
Forgiveness Region (mm)	1.00	1.00
Penalty Slope (voxel penalty/mm)	0.50	0.50
Max Distance Threshold (mm)	10.00	10.00

The table 3 presents a comparison of two sets of measurements, "pre-intervention" and "post-intervention", pertaining to ROIs 1 and 2, alongside relevant evaluation metrics for radiotherapy contouring. The data is described as follows:

ROI 1 and ROI 2 volumes: The volumes of the two ROIs are presented in cubic centimeters (cc) for both the "pre-intervention" and "post-intervention" scenarios. ROI 1, which is the expert's delineation of the cochlea, maintains a consistent volume of 0.14 cc, whereas ROI 2, which is the author's contour, exhibits a slight increase from 0.12 cc to 0.15 cc.

Matching, extra, and missing volumes: The matching volume, representing the overlap between the expert contour and the user-delineated structure, increases from 0.10 cc to 0.12 cc. The extra volume, which refers to the delineated volume outside the expert contour, decreases from 0.03 cc to 0.02 cc. The missing volume, corresponding to the undelineated portion of the expert contour, decreases from 0.04 cc to 0.02 cc.

Forgiveness metrics: The forgiven volumes for extra and missing portions are presented separately. For the extra volume, forgiven values decrease from 0.03 cc to 0.02 cc, while forgiven values for the missing volume increase from 0.01 cc to 0.02 cc.

Dice coefficient: This metric reflects the accuracy of the delineated structure to the expert's delineation. An increase from 0.73 to 0.86 indicates enhanced overlap between the structures.

StructSure™ score: This sensitive metric gauges the overall quality of the delineated structure, with higher scores reflecting greater accuracy. The score improves significantly from 88.45 to 99.85.

Forgiveness region, penalty slope, and maximum distance threshold: These parameters remain constant in both scenarios. The forgiveness region, which determines the acceptable deviation from the expert contour, is set at 1.00 mm. The penalty slope, representing the voxel penalty per millimeter, is set at 0.50. Lastly, the maximum distance threshold, beyond which the penalty is maximized, is established at 10.00 mm.

Overall, the table demonstrates improvements in contouring accuracy and delineation quality in the "post-intervention" scenario, which is the implementation of the window width and window level compared to the "pre-intervention" scenario, as evidenced by the increased Dice coefficient and StructSure™ score.

**Figure 3:**
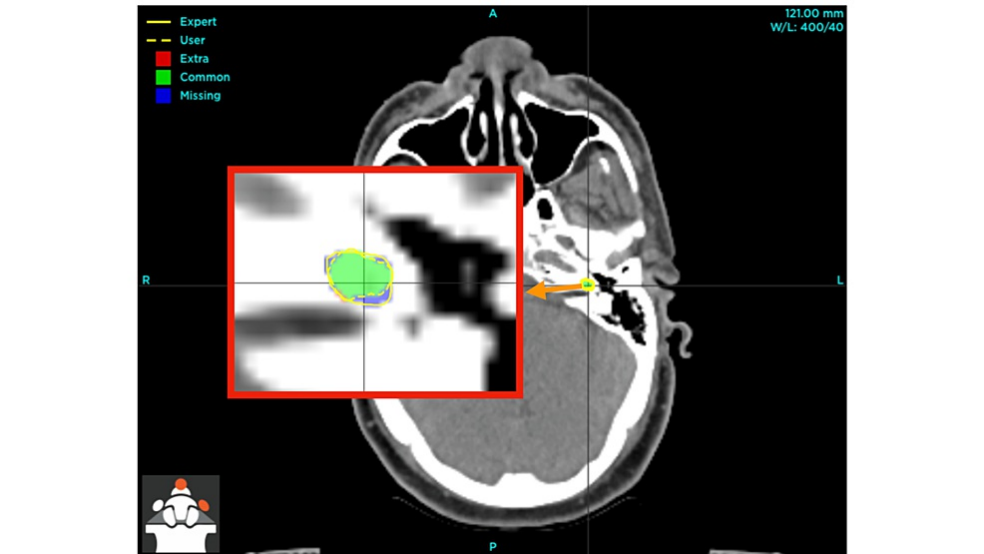
Pre-intervention discrepancies in left cochlea delineation The inset, indicated by an orange arrow, displays an enlarged perspective of the left cochlea, highlighting both the user (author) and expert contours, as well as the regions of overlap and discrepancy between the two delineations [10].

**Figure 4:**
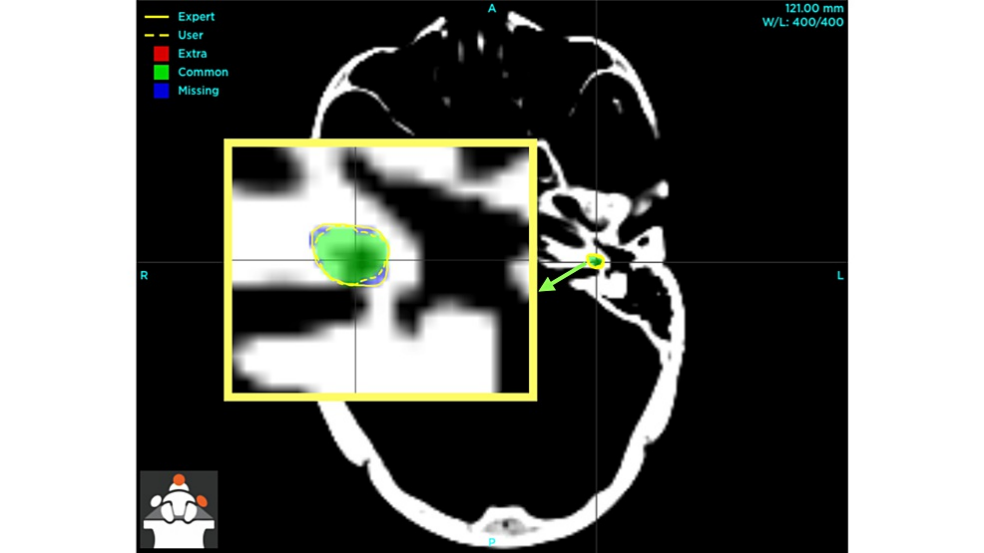
Post-intervention improvement in left cochlea visualization with optimized window width and level The inset, indicated by a green arrow, displays an enlarged perspective of the left cochlea, highlighting both the user (author) and expert contours, as well as the regions of overlap and discrepancy between the two delineations [10].

The discrepancies are discernible before implementing the specified intervention (Figure 3), and the refinement in the delineation of the left cochlea is evident after the application of the recommended step-by-step instructions, particularly the optimization of window width and window level to accurately visualize the hypodense area situated anterior to the internal auditory meatus (Figure 4).

 

Author Compared to the Population

A total of 863 participants (including the author) registered and executed the delineation exercise. We do not know the technique used by the 862 participants for the initial contours and later contours. As in the author's case, the initial contours (pre-intervention) were delineated with a window width of 400 and a window level of 40, while the later contours (post-intervention) were delineated after the implementation of the step-by-step instructions depicted in this study. The table presents a comparison of pre-and post-intervention StructSure and Dice scores for both the group and the author. The StructSure score measures the accuracy of contouring structures in radiation therapy planning, while the Dice score assesses the similarity between two sets of contours. Following the intervention, both the group and the author exhibit significant improvements in their respective StructSure and Dice scores. Notably, the author consistently demonstrates higher contouring accuracy and greater similarity to expert contours compared to the group's mean scores, both pre-and post-intervention (Table 4).

**Table 4:** A comparison of Dice and StructSure scores between the author and the group [10]

Metric	Group Pre-Intervention	Group Post-Intervention	Author Pre-Intervention	Author Post-Intervention
StructSure Score	31.85 (mean)	88.30 (mean)	88.45	99.85
Dice Score	0.28 (mean)	0.72 (mean)	0.729	0.861

## Discussion

The results of this study demonstrate that implementing step-by-step instructions, including adjustments to the window width and window level, can significantly improve contouring accuracy. The increased accuracy is evident in the post-intervention StructSure and Dice scores for both the right and left cochlea delineations. This improvement aligns with previous research findings highlighting the importance of standardized delineation protocols and their positive impact on contouring accuracy in radiotherapy planning [15].

The author's performance consistently surpassed the group's mean scores in both StructSure and Dice metrics, indicating a higher level of contouring accuracy and greater similarity to expert contours. This observation emphasizes the potential for individual variation in contouring proficiency, which may be influenced by factors such as experience, training, and familiarity with anatomical structures [16].

The Dice coefficient is a valuable tool for evaluating the degree of overlap between the contoured and expert standard structures. The increase in the author's and the group's Dice coefficients post-intervention indicates an improvement in the delineation of the cochlea, with the author consistently outperforming the group average.

The StructSure™ score, as a highly sensitive metric, allows for a thorough evaluation of the delineated structure's quality [11]. The higher the StructSure™ score, the more accurate the contours are compared to the expert contour. The post-intervention improvements in StructSure™ scores further support the effectiveness of the instructional technique in enhancing the accuracy of cochlea delineation.

The p-values (one-sample t-tests) indicate that there is a statistically significant difference between the author's scores and the group's mean scores, both for Dice (p<0.0001) and StructSure (p<0.0001) scores, and both pre-and post-intervention. The author's scores were higher than the group's mean scores, implying that the author performed better than the average of the group before the intervention. This was true for both right and left cochlear delineation.

A key aspect of the intervention in this study involved the adjustment of window width and window level settings. Previous research has shown that optimizing these parameters can enhance the visualization of anatomical structures, thereby improving contouring accuracy [17]. This study supports these findings, as the improved scores suggest that the author was better able to delineate the cochlea following the intervention.

In addition to the points discussed above, it is important to emphasize the potential clinical implications of improved cochlea delineation in radiotherapy contouring. Accurate delineation of organs at risk (OARs) is a crucial step in radiation therapy planning, as it directly impacts the dose distribution and, consequently, the potential side effects and treatment outcomes [18]. In the case of the cochlea, precise contouring is particularly important, as the organ is responsible for hearing and is sensitive to radiation-induced damage [19,20].

Moreover, the results of this study may also have implications for the training and education of radiation oncologists and radiation therapists. The step-by-step instructional technique presented here can be incorporated into training curricula and continued education programs to ensure that practitioners are equipped with the skills and knowledge required for accurate OAR delineation. The consistent improvement in contouring accuracy observed in both the author's and the group's performances after implementing the instructional technique highlights its potential value in a broader educational context.

Furthermore, the study's findings contribute to the growing body of literature emphasizing the importance of utilizing advanced image-processing techniques in radiotherapy contouring [21]. Future research could explore the integration of artificial intelligence and machine learning algorithms in the delineation process, as these technologies have shown promising results in automating and improving the accuracy of OAR delineation [22].

Limitations

Lack of Information on Participants' Techniques

The study does not provide information on the specific techniques used by the participants for their initial and later contours. This lack of information makes it difficult to assess the impact of different techniques on the overall results.

Limited Sample Size

Although the study includes a relatively large number of participants, increasing the sample size would have strengthened the findings by providing more robust and representative data.

Potential Selection Bias

Participants who registered and executed the delineation exercise might have been more motivated or skilled than the broader population of practitioners, which could lead to an overestimation of the intervention's effect on contouring accuracy and quality.

No Control Group

The study does not include a control group that did not receive the intervention (step-by-step instructions). This absence of a control group makes it difficult to determine the extent to which the improvements observed in the post-intervention data are directly attributable to the intervention. The comparison of the author's improvement to the participant's improvement and the calculation of the p-value have limitations as they were based on one sample t-test.

Generalizability

The study focuses on the delineation of cochlea structures in radiation therapy planning. The results may not be directly applicable to other structures or treatment planning scenarios, limiting the generalizability of the findings.

## Conclusions

In conclusion, the implementation of a step-by-step instructional approach focusing on the adjustment of window width and window level substantially enhanced the precision of cochlea delineation in radiotherapy contouring. The findings of this investigation may provide a foundation for future research concerning the optimization of contouring methodologies and the potential dosimetric ramifications of imprecise delineation in radiation therapy planning. The observed improvements in cochlea delineation accuracy bear significant clinical ramifications, encompassing the potential mitigation of radiation-related adverse effects and the augmentation of patient outcomes. Moreover, these results advocate for the incorporation of the step-by-step instructional technique into the training and education of radiation oncology practitioners and encourage further examination of advanced imaging processing techniques and artificial intelligence applications in radiotherapy contouring.
